# DNA Methylation Contributes to the Differential Expression Levels of *Mecp2* in Male Mice Neurons and Astrocytes

**DOI:** 10.3390/ijms20081845

**Published:** 2019-04-14

**Authors:** Vichithra R.B. Liyanage, Carl O. Olson, Robby M. Zachariah, James R. Davie, Mojgan Rastegar

**Affiliations:** 1 Regenerative Medicine Program, Max Rady College of Medicine, Rady Faculty of Health Sciences, University of Manitoba, Winnipeg, MB R3E 0J9, Canada; vichy.mb@gmail.com (V.R.B.L.); carl.olson@umanitoba.ca (C.O.O.); robbymz@gmail.com (R.M.Z.); 2 Department of Biochemistry and Medical Genetics, Max Rady College of Medicine, Rady Faculty of Health Sciences, University of Manitoba, Winnipeg, MB R3E 0J9, Canada; jim.davie@umanitoba.ca

**Keywords:** Rett syndrome, neurodevelopmental disorders, DNA methylation, epigenetics, MeCP2 isoforms, neurons, astrocytes, brain cells

## Abstract

Methyl CpG binding protein-2 (MeCP2) isoforms (E1 and E2) are important epigenetic regulators in brain cells. Accordingly, MeCP2 loss- or gain-of-function mutation causes neurodevelopmental disorders, including Rett syndrome (RTT), *MECP2* duplication syndrome (MDS), and autism spectrum disorders (ASD). Within different types of brain cells, highest MeCP2 levels are detected in neurons and the lowest in astrocytes. However, our current knowledge of *Mecp2*/MeCP2 regulatory mechanisms remains largely elusive. It appears that there is a sex-dependent effect in X-linked MeCP2-associated disorders, as RTT primarily affects females, whereas MDS is found almost exclusively in males. This suggests that *Mecp2* expression levels in brain cells might be sex-dependent. Here, we investigated the sex- and cell type-specific expression of *Mecp2* isoforms in male and female primary neurons and astrocytes isolated from the murine forebrain. Previously, we reported that DNA methylation of six *Mecp2* regulatory elements correlated with *Mecp2* levels in the brain. We now show that in male brain cells, DNA methylation is significantly correlated with the transcript expression of these two isoforms. We show that both *Mecp2* isoforms are highly expressed in male neurons compared to male astrocytes, with *Mecp2e1* expressed at higher levels than *Mecp2e2*. Our data indicate that higher DNA methylation at the *Mecp2* regulatory element(s) is associated with lower levels of *Mecp2* isoforms in male astrocytes compared to male neurons.

## 1. Introduction

The X-linked methyl CpG binding protein 2 (*MECP2/Mecp2*) gene encodes for two MeCP2 protein isoforms, E1 and E2. MeCP2 is a major transcriptional regulator in the brain with solid links to neurodevelopmental disorders [1]. Altered expression and function of *MECP2*/*Mecp2*/MeCP2 have been linked to Rett syndrome (RTT) [2,3,4], *MECP2* duplication syndrome (MDS) [5,6,7], autism spectrum disorders (ASD) [8,9], and fetal alcohol spectrum disorders (FASD) [10,11]. MeCP2-associated diseases have a strong sex correlation (especially in the case of RTT and MDS), possibly due to the X-linked nature of the *MECP2* gene [12] and/or X chromosome inactivation (XCI) in females [13]. Currently, these disorders have no cure, and restoring the normal MeCP2 levels is suggested as a possible therapeutic approach. Therefore, a better understanding of *Mecp2*/MeCP2 regulation in brain cells is critically important. Here, we aimed at studying the sex-specific levels of *Mecp2* isoforms in the two major cell types of the brain, namely neurons and astrocytes.

Originally, research on MeCP2 was mostly focused in neurons, primarily due to the belief that symptoms resulting from *Mecp2* loss of function were of neuronal origin. However, the discovery of MeCP2 expression in glia, and the functional consequences of glial *Mecp2* loss on neuronal phenotypes, caused a paradigm shift in the MeCP2 field [14]. Independent groups, including our team, have shown that total *Mecp2*/MeCP2 and MeCP2E1 expression are higher in neurons than in astrocytes [15,16]. We have shown differential expression of *Mecp2* isoforms during neural stem cell differentiation [17,18] and in the adult brain regions that have different cellular composition [19]. However, it is still unclear whether the expression of *Mecp2* isoforms in embryonic brain cell types is sex-dependent.

We have characterized DNA methylation of six regulatory elements (REs) in the *Mecp2* promoter (R1–R3) and intron 1 (R4–R6) that may impact *Mecp2* isoform-specific expression [17]. DNA methylation at these REs was correlated with *Mecp2* isoforms in different adult mouse brain regions [19], the embryonic forebrain [20], and alcohol-mediated change in *Mecp2* isoforms [18]. Hypermethylation of the *MECP2* promoter has also been detected in ASD patients with reduced *MECP2* levels [8,9], where there is a higher sex-association towards males (3 to 4 times higher in males compared to females) [21,22]. Therefore, correlational studies of DNA methylation at the *Mecp2* REs with *Mecp2* isoform-specific expression were done in male brain cells. Our results highlight the existence of a cell type-specific DNA methylation pattern at the *Mecp2* gene locus in male neurons and male astrocytes.

## 2. Results

### 2.1. Establishment of Sex-Specific Cultures of Male and Female Primary Neurons and Astrocytes

**Figure 1:**
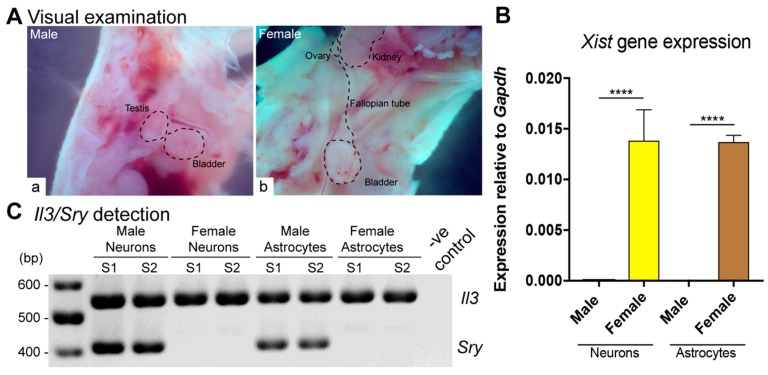
Culturing sex-specific neurons and astrocytes and their molecular identification. (**A**) Visual examination-based separation of male (a) and female (b) embryos based on observation of testes and ovaries, respectively. (**B**) Confirmation of sex-specific nature of cultured neurons and astrocytes using female-specific *Xist* gene expression. *Xist* transcript expression is detected only in female cells. For all samples, *N* = 3, except for female neurons, for which *N* = 2. Error bars represent standard error of the mean (SEM) with **** *p* < 0.0001. (**C**) *Il3/Sry*-based confirmation of male or female sex using agarose gel electrophoresis. PCR product sizes are *Sry*, 402 bp; *Il3*, 544 bp. *Sry* is detected only in males, while *Il3* is detected in all samples.

To characterize the cell type-specific expression of *Mecp2* isoforms in both sexes, we isolated and cultured male and female primary embryonic cortical neurons and astrocytes according to our previously established and validated protocols [15,23,24]. Separation and confirmation of embryonic sexes were performed by independent and parallel techniques. First, the male and female embryos were separated based on visual observation of testes in male embryos and fallopian tubes and ovaries in female embryos, respectively (Figure 1A). The sex of cultured cells was further confirmed by qRT-PCR-based detection of *Xist* transcripts, which are specific to females [25]. As expected, *Xist* transcripts were absent in male cells and were exclusively detected in female cells (Figure 1B). PCR-based detection of *Il3/Sry* genes was used to confirm the sex of male cells. *Sry* is expressed in male cells [26], while *Il3* is expressed in male and female cells [27]. Male cells showed detection of both *Sry* and *Il3*, thereby confirming the male origin of these cells (Figure 1C).

### 2.2. Mecp2 Isoforms Show Cell Type- and Sex-Specific Expression in Neurons and Astrocytes

**Figure 2:**
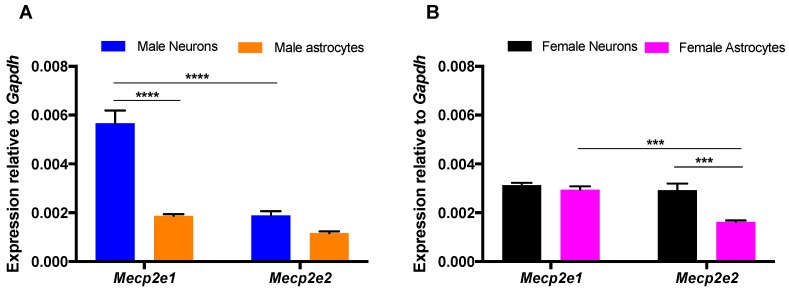
Cell type- and sex-specific expression of *Mecp2* isoforms in primary mouse embryonic neurons and astrocytes. The figure illustrates cell type-specific and sex-specific comparison of *Mecp2* isoforms in neurons and astrocytes. (**A**) *Mecp2* expression in male neurons and astrocytes. (**B**) *Mecp2* expression in female neurons and astrocytes. Significant differences are indicated with **** *p* < 0.0001, *** *p* < 0.001. For male neurons, male astrocytes, and female astrocytes, *N* = 3 ± SEM and *N* = 12 ± SEM (biological replicates are reported as *N,* while technical repeats of biological replicates are reported as *N*). For female neurons, *N* = 2 ± SEM and *N* = 8 ± SEM. The transcript levels were normalized to the endogenous control *Gapdh*.

*Mecp2*/MeCP2 expression level in brain cells is cell type-dependent [15,16]. *Mecp2* is X-linked and undergoes XCI escape with biallelic expression as early as the two-cell stage of mouse embryonic development [28]. Therefore, we determined *Mecp2e1* and *Mecp2e2* transcript levels in male neurons and male astrocytes and compared these levels to female cells (Figure 2).

Cell type-specific expression of *Mecp2e1* was 3.02-fold (*p* < 0.0001) higher in male neurons compared to male astrocytes (Figure 2A). Similarly, *Mecp2e2* transcripts were 1.63-fold higher in male neurons than in male astrocytes. This suggested that both *Mecp2* isoforms have higher expression in male neurons compared to male astrocytes. This pattern was not seen in female cells, and *Mecp2e1* transcript levels were similar for female neurons and astrocytes (1.06-fold: female neurons vs. female astrocytes). However, *Mecp2e2* transcript levels were higher (1.80-fold) in female neurons in contrast to female astrocytes (*p* < 0.001; Figure 2B). In male neurons, *Mecp2e1* transcripts were higher than *Mecp2e2* (2.99-fold, *p* < 0.0001; Figure 2A). Similarly, in male astrocytes, *Mecp2e1* levels were slightly higher than *Mecp2e2* (1.61-fold). In female neurons, *Mecp2e1* and *Mecp2e2* levels were similar (1.07-fold; Figure 2B), but *Mecp2e1* levels were higher than *Mecp2e2* in female astrocytes (1.80-fold, *p* < 0.001). Therefore, in both cell types, except for the female neurons, *Mecp2e1* transcripts were the major transcripts.

These observations provide evidence that these two studied brain cell types (neurons and astrocytes) have differential expression of *Mecp2* isoforms depending on the sex of embryos, raising the possibility that the transcription levels of these two isoforms are differentially regulated.

### 2.3. DNA Methylation at the Mecp2 Regulatory Elements May Contribute to Higher Expression of Mecp2 in Male Neurons Compared to Male Astrocytes with Lower Mecp2 Expression

**Figure 3:**
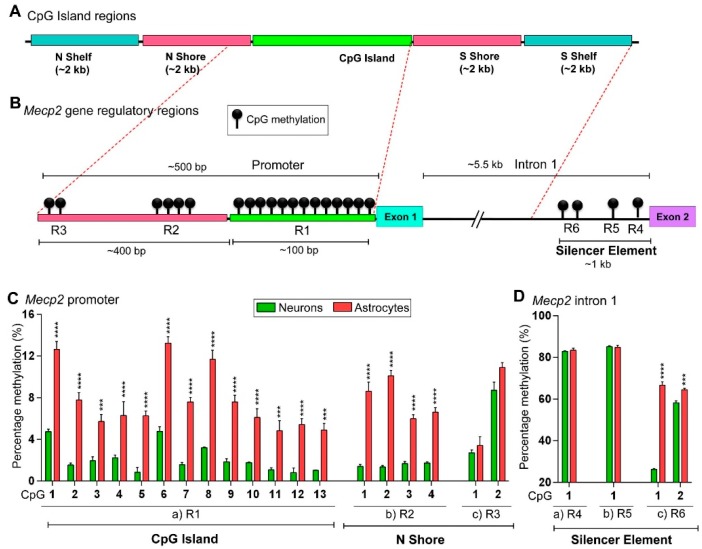
Bisulfite pyrosequencing analysis of DNA methylation at the *Mecp2* regulatory elements (REs) in male neurons and male astrocytes. (**A**) Schematic representation of CpG island regions characterized based on distribution of CpGs. Part A of the figure is modified from [34,35] (not drawn to scale). (**B**) Location of CpGs in *Mecp2* gene REs. Figure not drawn to scale. The figure illustrates the six previously reported REs [17], R1–R3 promoter Res, and R4–R6 intron 1 silencer element REs (not drawn to scale). The methylation symbol indicates the number of CpGs found within each RE: R1: 13 CpGs, R2: 4 CpGs, R3: 3 CpGs, R4: 1 CpG, R5: 1 CpG, and R6: 2 CpGs. (**C**) Percentage of DNA methylation (%) of CpG sites within the *Mecp2* promoter regions (a) R1, (b) R2, and (c) R3. (**D**) Percentage of DNA methylation (%) of CpG sites within the *Mecp2* intron 1 regions (a) R4, (b) R5, and (c) R6. Significant differences between male neurons and male astrocytes are indicated with **** *p* < 0.0001, *** *p* < 0.001, *N* = 3 ± SEM.

In a previous section, we showed the higher transcript levels of *Mecp2* isoforms in male neurons compared to male astrocytes, which was highly significant for *Mecp2e1*. To study if DNA methylation impacts the detected difference in *Mecp2e1* and *Mecp2e2* transcript levels, we analyzed male neurons and male astrocytes that contain only one X-chromosome, avoiding complications of X-linked inactivation that exists in female cells. For these studies, we analyzed DNA methylation patterns at the known *Mecp2* REs [17,18] (Figure 3B). Previous genome-wide DNA methylation analyses have indicated differential DNA methylation at the CpG islands that include the CpG islands, N shore, N shelf, S shore, and S shelf that flank the CpG islands (Figure 3A,B). The ‘shores’ comprise the 2-kb region flanking CpG islands and display dynamic DNA methylation patterns in contrast to the lower DNA methylation levels seen at CpG islands. The ‘shelves’ are located 4 kb further away from the CpG islands. Similar to shores, these regions are differentially methylated [29,30,31].

**Figure 4:**
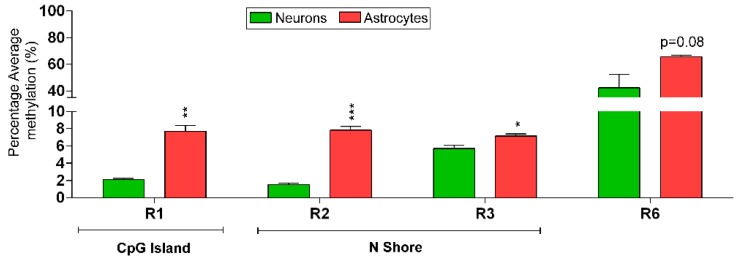
Average DNA methylation patterns over the *Mecp2* regulatory elements (REs) in male neurons and male astrocytes. Average percentage DNA methylation of all CpG sites in the promoter regions R1 (13 CpGs), R2 (4 CpGs), R3 (2 CpGs), and R6 of the intron 1 region (2 CpGs), in male neurons and male astrocytes. Significant difference between male neurons and male astrocytes is indicated with *** *p* < 0.001, ** *p* < 0.01, or * *p* < 0.05. *N* = 3. Error bars indicate standard error of the mean (SEM).

These previous studies suggest that hypomethylated or hypermethylated blocks of CpGs such as CpG islands or CpG shores may have a significant impact in determining the diagnosis of disease conditions such as cancer [31,32,33]. Also, increased methylation of the *MECP2* promoter in autistic male patients is reported to correlate with reduced *MECP2* expression [8]. Therefore, we analyzed DNA methylation at individual CpG sites (Figure 3C,D) and average DNA methylation over the entire REs (Figure 4). As reported previously [17,18], *Mecp2* promoter regions R1, R2, and R3 contained 13, 4, and 2 CpG sites, respectively. The intron 1 silencer element contained three REs, namely R4, R5, and R6, with 1, 1, and 2 CpG sites, respectively (Figure 3B).

Based on a general repressive role of DNA methylation, we hypothesized that higher *Mecp2* expression in male neurons is associated with lower DNA methylation at the *Mecp2* REs, while lower *Mecp2* expression in male astrocytes is associated with higher DNA methylation. First, we assigned the CpG island regions illustrated in Figure 3A to *Mecp2* gene REs (Figure 3B). R1 of *Mecp2* promoter is considered as a portion of a CpG island, which spans over exon 1. On the other hand, R2 and R3 belong to the N shore (Figure 3B). As intron 1 REs are located more than 4 kb downstream of the CpG island, they do not belong to either an S shelf or S shore. Based on previous studies in autistic patients and a rare case of an RTT patient [8,9,36], we hypothesized that blocks of DNA methylation at the CpG island and/or N shore may have differentially methylated CpG blocks, which may contribute to differential *Mecp2* expression in male neurons and male astrocytes. Bisulfite pyrosequencing experiments showed that the percentage of DNA methylation at the 13 CpGs within the *Mecp2* promoter region R1 was higher in male astrocytes compared to male neurons (CpG1: +7.87%, CpG2: +6.23%, CpG3: +3.77%, CpG4: +4.08%, CpG5: +5.42%, CpG6: +8.46%, CpG7: +6.02%, CpG8: +8.49%, CpG9: +5.75%, CpG10: +4.33%, CpG11: +3.76%, CpG12: +4.60%, and CpG13: +3.86%) (Figure 3C). In general, DNA methylation (5mC) of regulatory elements is associated with decreased gene transcription; therefore, higher DNA methylation could lead to reduced gene expression [37]. Our results are consistent with this idea that increased DNA methylation at these CpG dinucleotides in male astrocytes may collectively contribute to the lower *Mecp2* levels in these cells. Similarly, the percentage of DNA methylation at the four CpG sites at the R2 region was significantly higher in male astrocytes compared to male neurons (CpG1: +7.22%, CpG2: +8.74%, CpG3: +4.32%, and CpG4: +4.88%). However, DNA methylation at R3 CpGs was not statistically significant (CpG1: +0.71% and CpG2: +2.18%). Regardless, the proximal *Mecp2* promoter, which contains R1 and R2, showed a higher percentage of DNA methylation in male astrocytes compared to male neurons, potentially contributing to the lower *Mecp2e1* and *Mecp2e2* levels in male astrocytes. These data imply that in contrast to R1 and R2, DNA methylation at R3 of the promoter may not play a significant role in differential expression of *Mecp2* isoforms in male neurons and male astrocytes.

We then studied the *Mecp2* intron 1 regions for DNA methylation changes (Figure 3D). The CpG sites of R4 and R5 were similarly methylated in male astrocytes and male neurons (difference: CpG1-R4: +0.54%, CpG1-R5: −0.36%). However, the two CpGs within R6 had a significantly higher percentage of DNA methylation in male astrocytes compared to male neurons (CpG1: +40.40% and CpG2: +6.29%). Since this DNA methylation pattern is in agreement with our previous studies highlighting their role in *Mecp2* regulation [17,18], it is likely that higher methylation at the R6 region may contribute to lower expression of *Mecp2* isoforms in male astrocytes compared to male neurons. It is possible that DNA methylation at the intronic R4 and R5 may not play significant roles in differential expression of *Mecp2* isoforms in male neurons and male astrocytes.

Furthermore, we calculated the average DNA methylation of the REs that contained more than one CpG site (R1, R2, R3, and R6). Both R1 (+5.59%) and R2 (+6.29%) showed greater than 5% DNA methylation change over the entire region (Figure 4).

Although individual CpG sites of R3 were not significantly changed, the average DNA methylation over promoter R3 was slightly higher (+1.44%, *p* < 0.05) in male astrocytes. The average DNA methylation over individual R4–R6 CpG sites was not statistically significant. However, R6 showed +23.34% (*p* = 0.08) higher average DNA methylation in male astrocytes than in male neurons. Also, average methylation over the intron 1 silencer element (R4–R6) showed +11.45% hypermethylation in male astrocytes compared to male neurons. In other words, the difference in average DNA methylation between male neurons and male astrocytes was higher in the CpG island (R1), while the difference was reduced further away from the exonic region (R3). However, the average methylation within the intron 1 REs was similar between the two cell types, implying that the distinct differential average methylation observed in CpG island regions was not significantly different in the analyzed R4–R6 intronic region. Overall, these analyses showed that male neurons have significantly lower DNA methylation, mainly at the *Mecp2* promoter REs, in contrast to male astrocytes. DNA methylation levels at the *Mecp2* REs may contribute to the epigenetic regulation of *Mecp2* isoforms in male neurons and male astrocytes.

### 2.4. Expression of Mecp2 Isoforms Correlates with DNA Methylation at the Mecp2 REs

**Figure 5:**
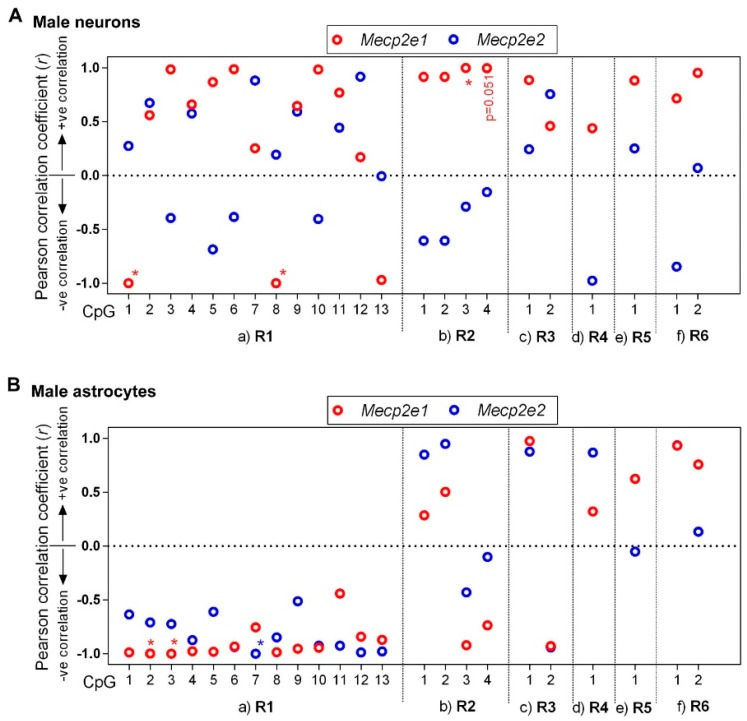
Correlation analyses between DNA methylation at the *Mecp2* regulatory elements (REs) and *Mecp2* isoform-specific expression in male neurons and male astrocytes. (**A**) Male neurons; (**B**) male astrocytes. All graphs represent the Pearson correlation coefficient (*r*) for *Mecp2e1* (red) and *Mecp2e2* (blue). Statistical significance of the correlation is indicated by * p < 0.05; *N* = 3. Individual CpG sites at the promoter regions (a) R1, (b) R2, and (c) R3 and intron 1 regions (d) R4, (e) R5, and (f) R6 are shown on the *x*-axis.

To gain insight into the impact of DNA methylation on *Mecp2e1* and *Mecp2e2* expression in male neurons and astrocytes, we performed a correlational analysis between *Mecp2* isoform-specific transcripts and DNA methylation at the individual CpG sites of R1–R6 (Figure 5). First, we determined the relation between *Mecp2e1* transcripts in male neurons with *Mecp2* promoter regions (Figure 5A). For R1, CpG1 showed a very strong negative correlation with *Mecp2e1* expression (*r* = −0.99, *p* < 0.05), implicating a repressive role of DNA methylation at this CpG. Similarly, CpG8 showed a very strong negative correlation with *Mecp2e1* (*r* = −0.99, *p* < 0.05). Although CpG13 showed a negative correlation with *Mecp2e1*, it was not statistically significant (*r* = −0.97, *p* = 0.15). The other CpG sites (CpG 2, 3, 4, 5, 6, 7, 9, 10, 11, and 12) showed varying degrees of positive correlations that were not statistically significant. Correlations between DNA methylation at the CpG sites of promoter R2 and *Mecp2e1* expression had a very strong positive nature (*r* > −0.9). CpG3 and CpG4 showed significant positive correlation (CpG3: *r* = +0.99, *p* < 0.05; CpG4: *r* = +0.99, *p* = 0.051), usually referring to an active role. *Mecp2e1* showed a positive but statistically insignificant correlation with DNA methylation at the promoter R3 and at the intron 1 regions R3, R4, and R6.

When correlation between *Mecp2e2* and DNA methylation at the REs was studied, we found that *Mecp2* REs showed varying degrees of positive and negative correlations. However, these correlations were statistically insignificant. It should be noted that this might not necessarily suggest the absence of a role for DNA methylation in *Mecp2e2* regulation in male neurons. This may be due to the involvement of different types of DNA methylation or that DNA methylation does not play a significant role here.

Unlike male neurons, which showed widely distributed correlation patterns between DNA methylation and *Mecp2* transcripts; male astrocytes demonstrated distinct DNA methylation patterns that correlated with *Mecp2* expression (Figure 5B). Both *Mecp2e1* and *Mecp2e2* expression in male astrocytes negatively correlated with DNA methylation of all CpGs of R1, which is in agreement with the repressive role of DNA methylation at R1 that we previously noted [17,18]. Among them, correlation between *Mecp2e1* and R1: CpG2 (*r* = −0.9984, *p* < 0.05) and R1: CpG3 (*r* = −0.9994, *p* < 0.05) was significant. Similarly, *Mecp2e2* showed a negative and significant correlation with DNA methylation at R1: CpG7 (*r* = −0.9999, *p* < 0.01). These data suggest that R1 may play a repressive role for both *Mecp2* isoforms. Correlation between DNA methylation at R2, R3, and intron 1 R4−R6 regions and *Mecp2* isoforms was statistically insignificant. However, R2: CpG1, R2: CpG2, and R3: CpG1 showed a positive correlation with both *Mecp2* isoforms. In contrast, R2: CpG3, R2: CpG4, and R3: CpG2 showed a negative correlation with both *Mecp2* isoforms. These data suggest that DNA methylation plays a different role in regulating *Mecp2* isoform-specific expression in male astrocytes and male neurons. Similar to neurons, DNA methylation at R4−R6 showed a positive but statistically insignificant correlation with *Mecp2e1*. Correlation between *Mecp2e2* and DNA methylation at R4−R6 was not fully consistent between the two cell types.

## 3. Discussion

Understanding how *Mecp2*/MeCP2 expression is controlled in major brain cell types is important not only developmentally, but also for human diseases affecting the brain. Such studies would provide insight in understanding how different levels of *Mecp2*/MeCP2 isoforms contribute to the maintenance of proper brain cellular functions and activities. Accordingly, investigation of *Mecp2* cell type-specific expression and regulation may provide valuable insights on how regulatory mechanisms can be used for potential strategies for MeCP2-associated diseases. Therefore, our study contributes to cell type- and sex-specific health research in brain cells.

Our study explored the expression of *Mecp2* isoforms in a sex-specific manner in primary neurons and astrocytes. The sex-specific difference in the basal level of *Mecp2* transcripts in embryonic brain cells highlights the importance of considering sex as a biological factor in MeCP2-associated neurodevelopmental disorders. In agreement with sex-specific levels of MeCP2, it is the study of *Mecp2*/MeCP2 expression in male and female rat brains during development [38] that indicated higher *Mecp2*/MeCP2 levels in the female amygdala and ventromedial hypothalamus at postnatal day 1 (P1) compared to male brain regions. However, at P10, males expressed more *Mecp2*/MeCP2 in the preoptic area. The observed higher expression of *Mecp2* in females than males in the rat brain at P1 is in agreement with the higher *Mecp2e1* levels in astrocytes and *Mecp2e2* levels in neurons and astrocytes. However, our study shows that in E18-isolated primary brain cells, *Mecp2e1* expression is higher in male than female neurons. In agreement with a previous report [38], our study demonstrates the possibility that *Mecp2* expression is sex-dependent, cell type-specific, and isoform-specific. These results may also implicate age/developmental stage-specific regulatory mechanisms.

Several studies have shown XCI escape and biallelic expression of the X-linked genes such as histone deacetylase 6 (*Hdac6*) and lysine demethylase 6A *(Kdm6a*) that are associated with the more accessible chromatin structure at the promoters of these genes, as indicated by DNase I hypersensitivity, occupancy of RNA polymerase phosphorylated at Ser 5 (PolII-S5p), recruitment of CCCTC-binding factor (CTCF), and enrichment of active histone modifications such as H3K4me3 [39,40]. Not only *MECP2*, but also other X-linked genes, such as GRB2-associated-binding protein 3 (*GAB3*), ribosomal protein S4, X-linked (*RPS4X*), Jumonji/ARID domain-containing protein 1C (*JARID1C*), ubiquitin-like modifier activating enzyme 1 (*UBE1*), baculoviral IAP repeat-containing protein 4 (*BIRC4*), and solute carrier family 16 member 2 (*SLC16A2*), show differential expression between the two sexes, with higher expression in female bovine fetal muscle tissues compared to males [41]. Generally, when genes escape XCI in females, they show higher expression in females due to biallelic expression, in contrast to monoallelic expression in males. However, here, the higher expression in females was not observed in all cases, suggesting such discordance regarding XCI escape could be due to other complicated regulatory mechanisms. Literature supports such discordance where male cells show higher gene expression. For instance, using microarray analysis, Talebizadeh et al. [42] demonstrated that only 10% of the 299 X-linked genes that they studied showed higher expression levels in the female human cerebrum (female (F) /male (M) ratio ≥ 0.7), and 17% of the genes showed higher X-linked gene expression in the male cerebrum (F/M ratio ≥ 1.5) [42]. The *MECP2* uni-gene cluster (Hs.200716) showed an F/M ratio = 0.8 in the cerebrum in this study, suggesting *MECP2* abundance in the male cerebellum may be higher than in the female cerebellum. The cerebellum is a brain region with the highest neuronal density in the brain [43,44]. Therefore, it is worth questioning whether there are deviating or complex *Mecp2* regulatory mechanisms seen in neurons, and more specifically, in male neurons, which drive higher *Mecp2* expression levels. However, in our study, the two *Mecp2* isoforms showed opposing trends (*Mecp2e1*: male > female; *Mecp2e2*: male < female). The presence of correlation between *Mecp2e1* and DNA methylation and the absence of such correlation with *Mecp2e2* sought us to speculate whether DNA methylation plays a role in this observation. It is also possible that an alternative splicing mechanism is shifted in male neurons in contrast to female neurons. In general, DNA methylation can regulate alternative splicing [10]. In our previous studies in differentiating murine neural stem cells, we proposed that DNA methylation status at R1 (promoter), R4, and R5 (intron 1 silencer element) might be involved in alternative splicing of the *Mecp2* gene. The potential impact of DNA methylation at the *Mecp2* intron 1 silencer element in regulating *Mecp2* splicing in neurons and astrocytes is also a possibility. However, investigating the role of DNA methylation in regulating *Mecp2* alternative splicing was not within the scope of this current study. Regardless, it is important to determine the role of DNA methylation in *Mecp2* alternative splicing, as this may modulate the expression of *Mecp2e1* and *Mecp2e2* in different type of brain cells including neurons and astrocytes. It is possible that a ‘kinetic coupling model’ of cotranscriptional splicing *via* DNA methylation- and CTCF-mediated mechanisms [45] may contribute to *Mecp2* splicing in neurons and astrocytes. Regardless, establishing the precise role of DNA methylation in *Mecp2* splicing warrants further investigations.

Here, DNA methylation at the six *Mecp2* REs was determined by bisulfite pyrosequencing, followed by correlational analysis in male neurons and male astrocytes. While comparison between male neurons and male astrocytes highlighted a negative correlation between *Mecp2* expression and DNA methylation, correlational analysis in male neurons showed otherwise for *Mecp2* promoter R2. Generally, bisulfite pyrosequencing does not distinguish between 5mC and 5hmC DNA methylation marks [46] and may somehow overrepresent 5mC [47]. Therefore, additional analysis of 5mC and 5hmC at the *Mecp2* REs may provide further insight. Within the body, the brain and specifically neurons show the highest conserved non-CpG DNA methylation [48], which could provide more in-depth knowledge. We observed that the strongest correlation to be in the R1 promoter region between male neurons and male astrocytes, which was relatively small in terms of the percentage of DNA methylation. However, as we reported for *Mecp2* in differentiating brain-derived embryonic neural stem cells, even small percentage changes at its REs might be biologically significant and cause 2–3-fold change at the MeCP2 protein levels [17]. In general, DNA methylation may also exist in the form of CpH (rather than CpG) and may be differently involved in primary neurons and astrocytes. Although CpH DNA methylation was not part of our reported study here, investigation of CpH DNA methylation would be important in the context of cell type-specific expression of *Mecp2e1*/*e2*.

Previously, we reported differential correlation of *Mecp2* isoforms with DNA methylation that varied with the stages of neural stem cell differentiation [17] or between murine strains [20]. For differentiating neural stem cells isolated from E14 mouse embryos, the correlation was seen in almost all the CpG sites with both *Mecp2* isoforms. However, in *in vivo* differentiated neurons isolated from E18 embryos in this study, significant correlation was limited to four CpGs in the *Mecp2* promoter and with *Mecp2e1*. Similarly, the number of significant correlations observed in male astrocytes isolated from E18 embryos was low, albeit the correlations that were observed for both *Mecp2* isoforms. This may implicate that DNA methylation might be more critical at earlier stages of embryonic development and during neural stem cell differentiation, in comparison to differentiated cell types at later stages of embryonic development. These differences further emphasize the existence of cell type-specific *Mecp2* regulatory mechanisms. Yet, differential cell type-specific expression and/or regulation make it challenging to develop strategies to rescue abnormal MeCP2 expression in disease conditions. For instance, decitabine treatment during embryonic neural stem cell differentiation induced *Mecp2e1*/MeCP2E1 [17], while similar studies in fibroblasts isolated from RTT patients did not respond to 5-aza-2′-deoxycytidine (similar to decitabine) treatment at lower concentrations [49]. It is questionable as to whether similar treatments would be successful in elevating *Mecp2*/MeCP2 expression in differentiated cell types such as neurons and in later stages of development or adulthood. DNA-demethylating agents that are capable of inducing *Mecp2*/*MECP2*/MeCP2 levels may be helpful for RTT. However, it is unclear if knowledge on the effect of DNA methylation on *Mecp2* could be used in *MECP2* duplication syndrome. In lens epithelial cells, scientists demonstrated that a DNMT inhibitor (which can also be a DNA-demethylating agent), zebularine, attenuated MeCP2 expression in a time- and dose-dependent manner [50]. Similarly, in the human fetal retinal pigment epithelial cells, 5-aza-2′-deoxycytidine reduced MeCP2 expression in a dose-dependent manner [51]. Therefore, it is possible that depending on the cell type, the effect of DNA-demethylating agents and/or DNMT inhibitors on MeCP2 expression may be different.

## 4. Materials and Methods

### 4.1. Ethics

Our experimental studies were performed in agreement with the standards of the Canadian Council on Animal Care with approval of the Office of Research Ethics, University of Manitoba. All procedures were reviewed in advance of conducting the experiments and were approved by the University of Manitoba Protocol Management and Review Committee at the Bannatyne Campus, under the approved protocol number 12-031/1/2 (16 June 2014) and subsequent renewal(s).

### 4.2. Primary Culture of Embryonic E18.5 Neurons

Primary embryonic neurons from the cortex of E18.5 CD1 mice were isolated and cultured as previously described [15,23,24]. Embryos were separated based on the sex by visual sex recognition under a dissecting Zeiss microscope. In brief, dissected cortices were dissociated by papain and trituration through a Pasteur pipette. Dissociated cells were resuspended in neurobasal media supplemented with B27 and were then plated at a density of 1.2 × 10^5^ cells/mL in poly-lysine-coated dishes. Half of the media was replaced after 72 hours (h) and was refreshed every 48 h thereon. According to published protocols for primary neurons, cells were collected after eight days in culture.

### 4.3. Primary Culture of Embryonic E18.5 Astrocytes

Primary embryonic astrocytes from the cortex of E18.5 CD1 mice were cultured as previously described [15,24]. Embryos were separated based on the sex and as described above for primary neurons. In brief, dissected cortices were further dissociated using papain enzyme and triturated with Pasteur pipette. Cells were subsequently resuspended in minimum essential medium (MEM, Thermo Fisher Scientific) supplemented with 10% FBS. Cells were then seeded at a density of 2 × 10^5^ cells/mL in poly-lysine-coated dishes. Media were replaced every 48 h until the day of collection. According to the published protocols for primary astrocytes, cells were collected after 15 days.

### 4.4. Culture and Identification of Sex-Specific Neurons and Astrocytes

Genomic DNA was extracted from neurons and astrocytes by DNeasy Blood and Tissue Kit (Qiagen). Semiquantitative polymerase chain reaction (PCR)-based amplification of sex-determining region protein gene on Y chromosome (*Sry*) was done as previously reported [17] to identify and confirm male cells. We used the interleukin 3 (*Il3*) gene as an autosomal gene and an internal control for both sexes. PCR products were identified based on size (*Sry*, 402 base pairs (bp); *Il3*, 544 bp). RNA was extracted from male and female cells by Trizol extraction method (Life Technologies Inc., 15596-026) and mirVana RNA extraction kit (Thermo Fisher Scientific, AM1560), respectively. Quantitative reverse transcription PCR (qRT-PCR) for X-inactive specific transcripts (*Xist*) was done for female cells as previously described [17,52].

### 4.5. Quantitative RT-PCR (qRT-PCR)

RNA was extracted from male cells and female cells by Trizol and mirVana RNA extraction kit methods, respectively. RNA that was converted to cDNA using previously established protocols prior to qRT-PCR [17]. Relative gene expression was calculated with reference to the housekeeping gene glyceraldehyde 3-phosphate dehydrogenase (*Gapdh*).

### 4.6. DNA Methylation Analysis by Bisulfite Pyrosequencing

Bisulfite pyrosequencing was performed for the six *Mecp2* REs (R1 to R6) as a service at the Hospital for Sick Children, Toronto, Canada, as we reported previously [17,18,19,20]. For the sequence of the primers, please refer to our previous reports [17,18,19,20].

### 4.7. Correlation Analysis between Detected DNA Methylation at the Mecp2 Regulatory Elements and Transcript Expression Levels of Mecp2 Isoforms

Pearson’s correlation analysis and linear regression were done as we reported previously [17,19], and statistical significance was determined at *p* < 0.05 as described elsewhere [17,18,19].

### 4.8. Statistical Analysis

The graphs represent an average of 2-3 independent biological experiments (*N* = 2–3) and 8–12 technical replicates (*n* = 8–12) with error bars showing standard error of the mean (SEM). Statistical significance was determined at **** *p* < 0.0001, *** *p* < 0.001, ** *p* < 0.01, or * *p* < 0.05. All experiments were performed in primary cortical neurons or astrocytes isolated from 2–3 separate pregnant mothers as *N* = 2–3 biological replicates [17,18,24]. For the comparison of cell type-specific, sex-specific, and isoform-specific expression of *Mecp2* isoforms, two-way ANOVA was used.

## 5. Conclusions

**Figure 6:**
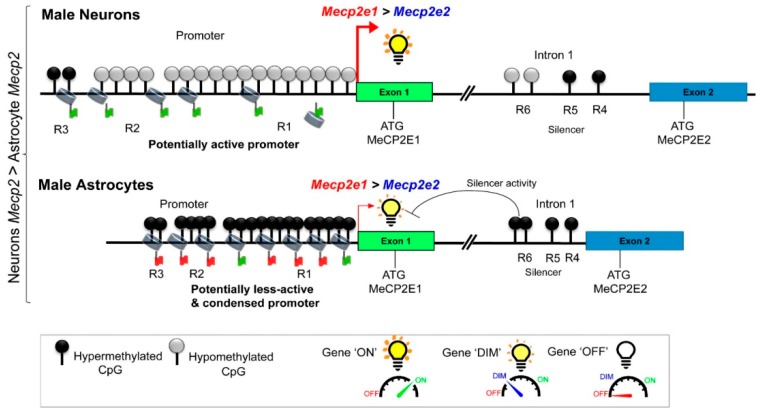
Proposed model for potential control of *Mecp2e1* and *Mecp2e2* by DNA methylation in male neurons and male astrocytes. Based on the findings of this study, this figure illustrates a summary of the proposed mechanism of *Mecp2* regulation by DNA methylation in male neurons and male astrocytes. Higher *Mecp2* expression levels in male neurons compared to male astrocytes could be in part driven by lower DNA methylation. It is possible that a lower DNA methylation at the *Mecp2* promoter in male neurons may induce an “active chromatin conformation”, allowing higher transcription. In contrast, higher DNA methylation at the *Mecp2* promoter in male astrocytes may cause a “less active and more condensed chromatin conformation”, leading to lower *Mecp2* level. We propose that DNA methylation may act as a switch for fine-tuning of the *Mecp2* gene expression from “ON” (active promoter) to “DIM” (less active promoter) to “OFF” (repressed promoter).

In conclusion, our studies presented here report the cell type-, sex-, and isoform-specific expression of *Mecp2* isoforms in primary cortical neurons and astrocytes. Neither *Mecp2* isoform showed equal expression in male and female cell types. In comparison to male astrocytes, male neurons had higher levels of *Mecp2* transcripts. Our results provide evidence that the DNA methylation of *Mecp2* regulatory elements contributes to the differential expression of *Mecp2* in male neurons and astrocytes. In male neurons, *Mecp2e1* was the major isoform, with approximately three-fold higher levels than *Mecp2e2*. The reduced methylation of the *Mecp2* response elements in male neurons may alter transcription elongation rates and splicing of the *Mecp2* transcripts, giving rise to greater levels of *Mecp2e1* transcripts. The major findings of our study and potential mechanisms are summarized in Figure 6.

## Abbreviations

5hmC	5-hydrocymethylcytosine
5mC	5-methylcytosine
ASD	autism spectrum disorder
BIRC4	baculoviral IAP repeat-containing protein 4
bp	base pairs
cDNA	complementary DNA
CTCF	CCCTC-binding factor
DNMT	DNA methyltransferase
E	embryonic day
ENCODE	Encyclopedia of DNA Elements
F	female
FASD	fetal alcohol spectrum disorder
FBS	fetal bovine serum
GAB3	GRB2-associated-binding protein 3
*Gapdh*	glyceraldehyde 3-phosphate dehydrogenase
h	hours
*Hdac*	Histone deacetylase 6
*Il3*	interleukin 3
JARID1C	Jumonji/ARID domain-containing protein 1C
*Kdm6a*	lysine demethylase 6A
M	male
MDS	*MECP2* duplication syndrome
MeCP2	Methyl CpG binding protein 2
P	postnatal day
PCR	polymerase chain reaction
PolII-S5p	RNA polymerase phosphorylated at Ser 5
qRT-PCR	quantitative reverse transcription PCR
R	region
r	Pearson’s correlation coefficient
REs	regulatory elements
RPS4X	ribosomal protein S4 X-linked
RTT	Rett syndrome
SEM	standard error of the mean
*SLC16A2*	solute carrier family 16 member 2
*Sry*	sex-determining region protein gene on the Y chromosome
UBE1	ubiquitin-like modifier activating enzyme 1
XCI	X chromosome inactivation
*Xist*	X-inactive specific transcript
